# Confirmation of *Tylototritonziegleri* Nishikawa, Matsui & Nguyen, 2013 in China, with discussion on the relationship between *T.verrucosus* Anderson, 1871 and *T.panwaensis* Grismer, Wood, Quah, Thura, Espinoza & Murdoch, 2019 (Caudata, Salamandridae)

**DOI:** 10.3897/BDJ.10.e82707

**Published:** 2022-05-19

**Authors:** Shuo Liu, Mian Hou, Dingqi Rao

**Affiliations:** 1 Kunming Natural History Museum of Zoology, Kunming Institute of Zoology, Chinese Academy of Sciences, Kunming, China Kunming Natural History Museum of Zoology, Kunming Institute of Zoology, Chinese Academy of Sciences Kunming China; 2 College of Continuing (Online) Education, Sichuan Normal University, Chengdu, China College of Continuing (Online) Education, Sichuan Normal University Chengdu China; 3 Kunming Institute of Zoology, Chinese Academy of Sciences, Kunming, China Kunming Institute of Zoology, Chinese Academy of Sciences Kunming China

**Keywords:** morphology, ND2, newt, phylogeny, Yunnan

## Abstract

**Background:**

The distribution of the Ziegler’s Crocodile Newt *Tylototritonziegleri* Nishikawa, Matsui & Nguyen, 2013 in China has been controversial. This species was originally recorded uncertainly from Guangxi Autonomous Region, China. Subsequently, this species was recorded from Yunnan and Guangdong provinces, China. Thereafter, the record from Guangdong was denied and the record from Yunnan was questioned.

**New information:**

Two specimens of *Tylototriton* Anderson, 1871 were collected from Wenshan Prefecture, Yunnan Province, China, in 2020. Phylogenetically, the sequences of these two specimens clustered with the sequences of *T.ziegleri* (including the holotype) from its type locality with strong support, and morphologically agree well with the original description of *T.ziegleri*. We confirm the record of *T.ziegleri* in China and present detailed collection site and morphological description of the specimens from China. In addition, we found that *T.panwaensis* Grismer, Wood, Quah, Thura, Espinoza & Murdoch, 2019 may be the synonym of *T.verrucosus* Anderson, 1871. We discussed the relationship between *T.verrucosus* and *T.panwaensis*.

## Introduction

*Tylototritonziegleri* Nishikawa, Matsui & Nguyen, 2013 is a member of the *T.asperrimus* species group, that was described from northern Vietnam (Nishikawa et al. 2013). This species was previously reported in China by Hernandez (2016) from Jingxi County, Guangxi Autonomous Region; however, he also mentioned that the population in Jingxi may not be the true *T.ziegleri*. Subsequently, this species was reported from Malipo County, Yunnan Province, based on only molecular data of a single specimen without morphological description and detailed collection information (Ye et al. 2017). Thereafter, this species was reported from Guangdong Province, China by Li et al. 2020. Lyu et al. (2021) found that the record of *T.ziegleri* from Guangdong by Li et al. (2020) actually represented a new species at that time and described it as *T.sini* Lyu, Wang, Zeng, Zhou, Qi, Wan & Wang, 2021. Therefore, Lyu et al. (2021) removed the record of *T.ziegleri* from the herpetofauna of Guangdong. In addition, Lyu et al. (2021) questioned the record of *T.ziegleri* from Yunnan.

*Tylototritonverrucosus* Anderson, 1871 is the type species of the genus *Tylototriton*. However, the current taxonomy of the true *T.verrucosus* is problematic because Anderson (1871) neither specified an exact type locality nor mentioned which specimens the original description of *T.verrucosus* was based on. *Tylototritonpanwaensis* Grismer, Wood, Quah, Thura, Espinoza & Murdoch, 2019 is another species of *Tylototriton* closely resembling *T.verrucosus* and this species was described from Kachin State, north-eastern Myanmar, a location close to the border between Myanmar and western Yunnan of China.

During our field surveys in south-eastern Yunnan, China, in 2020, two specimens of *Tylototriton* were collected from Malipo County, Wenshan Prefecture. Detailed morphological comparisons and molecular analysis indicated that these specimens belong to *T.ziegleri*. Therefore, we confirm the distribution of *T.ziegleri* in China and provide a detailed description of these specimens herein. In addition, we collected some specimens of *T.panwaensis* in 2019 from western Yunnan, China, which is probably the type locality of *T.verrucosus*. Therefore, we also discuss the relationship between *T.verrucosus* and *T.panwaensis* herein.

## Materials and methods

### Sampling

Specimens were collected, humanely euthanised and then fixed in 75% ethanol for permanent storage. Photographs were taken to document the colour pattern in life prior to euthanasia. Liver tissue samples were preserved in 99% ethanol for molecular analysis. All specimens were deposited at Kunming Natural History Museum of Zoology, Kunming Institute of Zoology, Chinese Academy of Sciences (KIZ).

### Morphological characteristics

Morphological terminology followed Nishikawa et al. (2013). Measurements were taken with an electronic vernier caliper to the nearest 0.1 mm. Morphometric characters included: SVL: snout-vent length, from tip of snout to anterior tip of vent; HL: head length, from tip of snout to wrinkle of throat; HW: head width, measured at the angle anterior to the parotid gland; MXHW: maximum head width, measured at widest point; SL: snout length, from tip of snout to anterior tip of upper eyelid; LJL: lower jaw length, from tip of lower jaw to jaw angle; ENL: eyelid-nostril length, minimum distance between eyelid and nostril; IND: internarial distance, minimum distance between the external nares; IOD: interorbital distance, minimum distance between upper eyelids; UEW: upper eyelid width, greatest width of upper eyelid; UEL: upper eyelid length, greatest length of upper eyelid; OL: orbit length, maximum length of orbit; AGD: axilla-groin distance, minimum distance between axilla and groin; TRL: trunk length, from wrinkle of throat to anterior tip of vent; TAL: tail length, from anterior tip of vent to tail tip; VL: vent length, from anterior to posterior tip of vent; BTAW: basal tail width, tail width measured at root of tail; MTAW: medial tail width, tail width measured at middle; BTAH: basal tail height, tail height measured at base of tail; MXTAH: maximum tail height, tail height measured at highest point; MTAH: medial tail height, tail height measured at middle; FLL: fore-limb length, distance from axilla to tip of longest finger; HLL: hind-limb length, 2FL: second finger length; 3FL: third finger length; 3TL: third toe length; and 5TL: fifth toe length.

### Molecular analysis

Genomic DNA was extracted from liver tissue samples preserved in 99% ethanol using a standard phenol-chloroform extraction protocol (Sambrook et al. 1989). A fragment of the NADH dehydrogenase subunit two (ND2) was amplified for the newly-collected specimens. Methods for amplification and sequencing of the DNA fragment are the same as Lyu et al. (2021). All new sequences were deposited in GenBank. Homologous and outgroup sequences were obtained from GenBank (Table 1).

### Phylogenetic analyses

Sequences were aligned using MUSCLE 3.6 (Edgar 2004) with default parameters. Average genetic distances were calculated in MEGA 11 (Tamura et al. 2021) using the uncorrected p-distance model. The best substitution model GTR+F+I+G4 was selected using the Akaike Information Criterion (AIC) in ModelFinder (Kalyaanamoorthy et al. 2017). Bayesian Inference was performed in MrBayes 3.2.7 (Ronquist et al. 2012). Two runs were performed simultaneously with four Markov chains, the chains were run for 10,000,000 generations and sampled every 1,000 generations, the first 25% of the initial samples was discarded as burn-in after the standard deviation of split frequencies of the two runs was less than a value of 0.01. Maximum Likelihood analysis was performed in IQ-TREE 1.6.12 (Nguyen et al. 2015). The ultrafast bootstrap approximation algorithm was used via 1,000 bootstrap pseudoreplicates.

## Taxon treatments

### Tylototriton ziegleri

Nishikawa, Matsui & Nguyen, 2013

37D8EA30-C450-5811-945F-44438064B7DB

#### Materials

- **Type status:** Other material. **Occurrence:** catalogNumber: KIZ20210504; individualCount: 1; sex: male; lifeStage: adult; **Taxon:** scientificName: *Tylototritonziegleri* Nishikawa, Matsui & Nguyen, 2013; family: Salamandridae; **Location:** country: China; stateProvince: Yunnan; locality: Zhongzhai Village, Xiajinchang Township, Malipo County, Wenshan Prefecture; verbatimElevation: 1750 m; verbatimCoordinates: 23°7′8″N 104°50′6″E; **Event:** eventRemarks: collected by Shuo Liu on 10 May 2020; **Record Level:** basisOfRecord: preserved specimen
- **Type status:** Other material. **Occurrence:** catalogNumber: KIZ20210505; individualCount: 1; sex: male; lifeStage: adult; **Taxon:** scientificName: *Tylototritonziegleri* Nishikawa, Matsui & Nguyen, 2013; family: Salamandridae; **Location:** country: China; stateProvince: Yunnan; locality: Zhongzhai Village, Xiajinchang Township, Malipo County, Wenshan Prefecture; verbatimElevation: 1750 m; verbatimCoordinates: 23°7′8″N 104°50′6″E; **Event:** eventRemarks: collected by Shuo Liu on 10 May 2020; **Record Level:** basisOfRecord: preserved specimen

#### Description

##### Description the specimens from China

**Figure 1.**
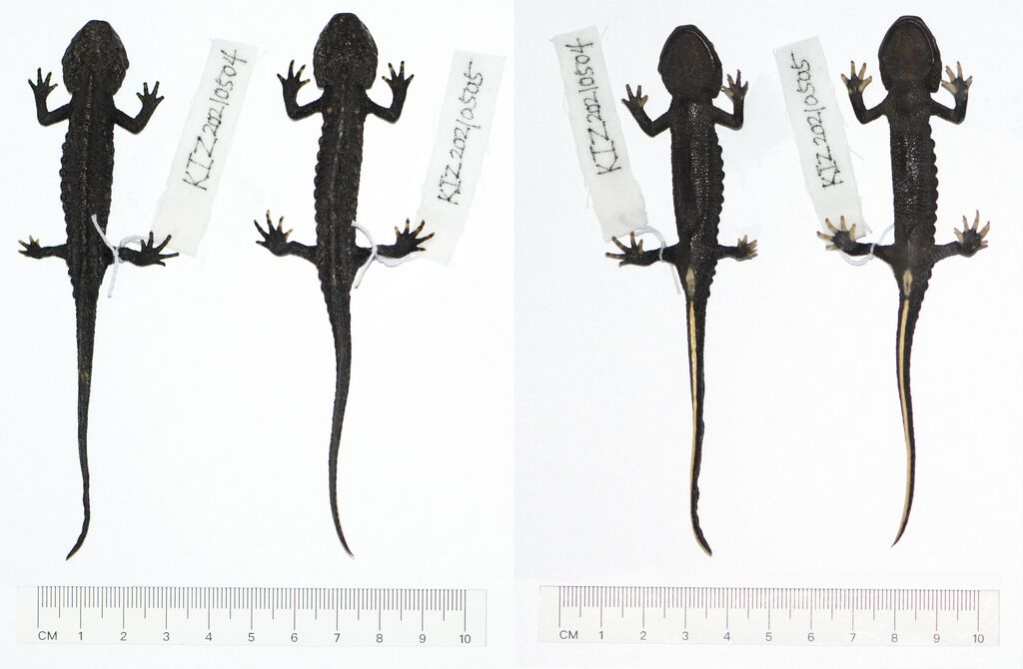
*Tylototritonziegleri* from China in preservative. Dorsal view (left) and ventral view (right).

Morphometric data are provided in Table 2. Body moderately stout, medium in size (SVL 58.2–60.8 mm, TAL 68.0–69.7 mm). Head width almost equal to head length (HW/HL 0.96–1.05); head nearly hexagonal in shape in dorsal view, depressed, gently sloping in profile. Snout short, truncate, slightly beyond lower jaw. Nostril on anterior margin of snout, located notably closer to snout tip than to eye. Tongue oval, not notched distally, attached to mouth floor, but free laterally; vomerine tooth series in an inverted V-shape, converging anteriorly, but not reaching choanae. Labial fold absent; gular fold present, but weak; parotoids distinct, projecting posteriorly; costal folds absent. Dorsolateral supratemporal bony ridges on head protruding, beginning at the anterior corner of orbit continuing to anterior end of parotoid, posterior ends curved inside; mid-dorsal bony ridge on head short. Vertebral mid-dorsal ridge distinctly protruding, segmented, forming a row of tubercles, running from occiput region to the base of tail, separated from mid-dorsal bony ridge on head by a small gap. Rib nodules distinct, forming knob-like warts, relatively small, arranged in two longitudinal series on dorsolateral surfaces of dorsum from axilla to base of tail, counting 15–17 nodules on each side of body; rib nodules in the middle largest and decreasing anteriorly and posteriorly (Fig. 1).

Limbs slender, tips of fore-limbs and hind-limbs overlapping when adpressed towards each other along body; fingers and toes free of webbing; relative finger lengths III ＞II ＞ I ≥ IV, relative toe lengths III ＞ IV ＞ II ＞ I ≥ V. Tail long, TAL/SVL 1.12–1.20; laterally compressed along entire length, tapering posteriorly, tip pointed, dorsal fin more distinct posteriorly, ventral ridge smooth.

Skin rough with fine granules, dense on dorsum and ventrum, but small and sparse on throat. Cloacal region slightly swollen, vent as a longitudinal slit, vent edges with numerous small transverse folds.

##### Colouration in life

**Figure 2.**
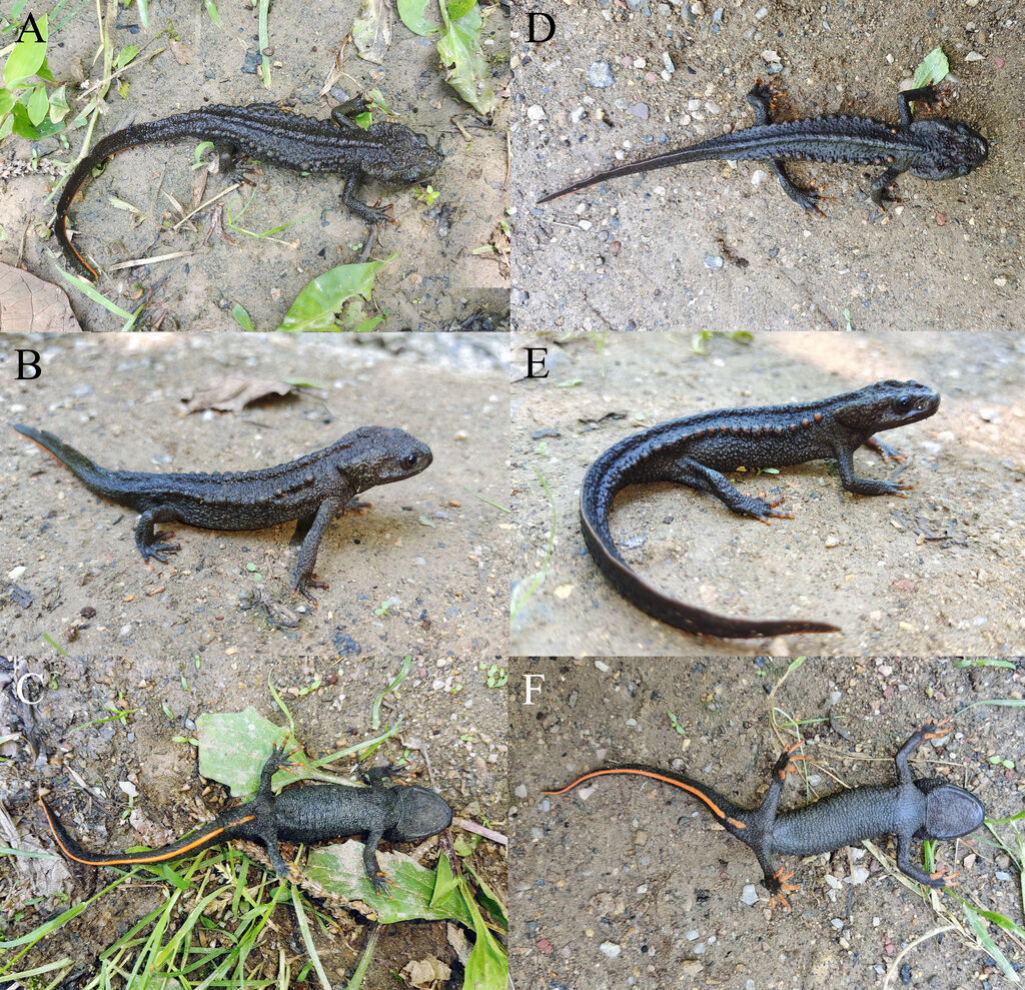
*Tylototritonziegleri* from China in life: **A-C** the adult male KIZ20210504; **D-F** the adult male KIZ20210505.

Dorsum almost uniformly black; venter slightly lighter than dorsum; bony ridges on head and vertebral ridge black, rib nodules black or reddish, only tips or most regions of fingers and toes, vent and ventral ridge of tail orange (Fig. 2).

##### Ecological notes

The specimens were collected in a small stream in the forest at night, the water in the stream was shallow, and both sides of the stream were covered with vegetation. No eggs or larvae were found.

##### Distribution

**Figure 3.**
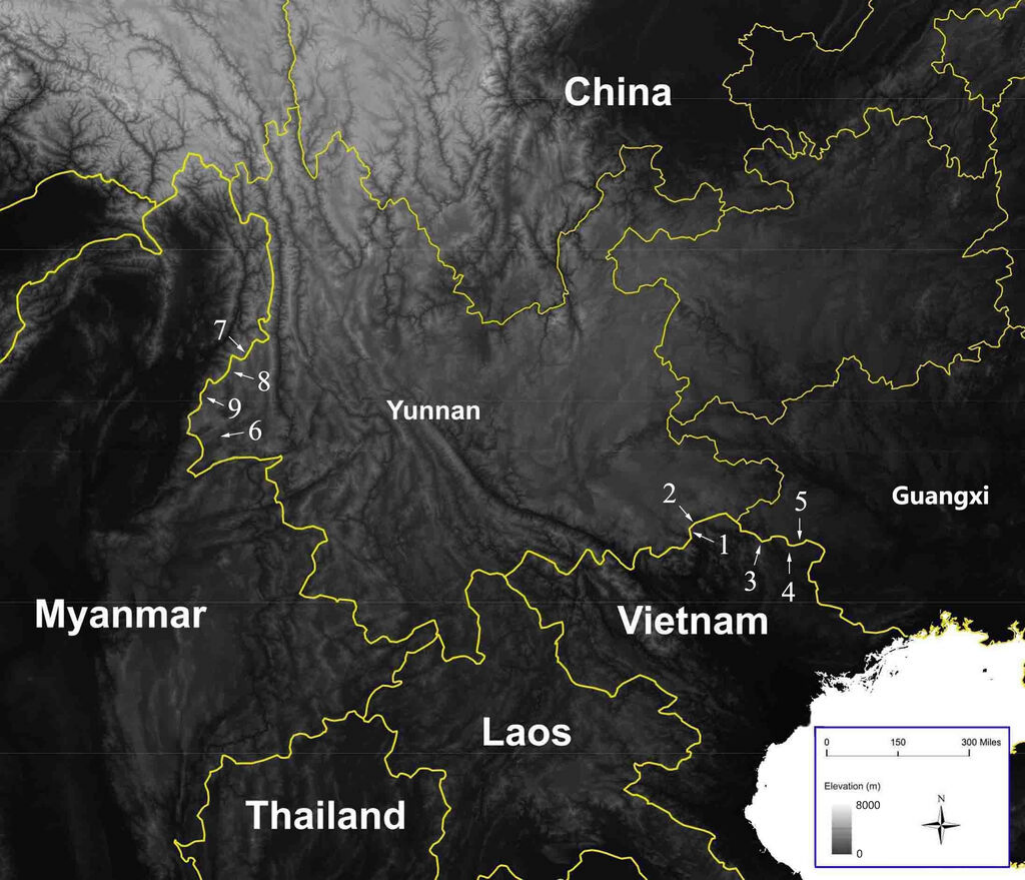
Map showing the type locality of *Tylototritonziegleri* in Ha Giang, Vietnam (1); the collection site of *T.ziegleri* in Malipo, Yunnan, China (2); the distributions of T.cf.ziegleri in Cao Bang, Vietnam (3 and 4); the distribution of T.cf.ziegleri in Jingxi, Guangxi, China (5); the type locality of *T.verrucosus* designated by Nussbaum et al. (1995) in Longchuan, Yunnan, China (6); the type locality of *T.panwaensis* in Kachin, Myanmar (7); the collection site of *T.panwaensis* in Tengchong, Yunnan, China (8); and the collection site of *T.panwaensis* in Yingjiang, Yunnan, China (9).

This species was recorded from Ha Giang and Cao Bang provinces, northern Vietnam; Malipo County, Yunnan Province and Jingxi County, Guangxi Autonomous Region, China (Fig. 3). However, the populations in Cao Bang and Jingxi may not represent the true *Tylototritonziegleri* (see Discussion section).

## Analysis

Morphologically, the specimens from Malipo County, Wenshan Prefecture, Yunnan, China, agree well with the original description of *Tylototritonziegleri*: medium body size; skin rough with fine granules; bony ridges on head distinct; vertebral ridge prominent and segmented; rib nodules prominent; tips of fore-limbs and hind-limbs overlapping when adpressed along body; tail thin; dorsum uniform blackish; finger and toe tips, vent and ventral ridge of tail orange.

**Figure 4.**
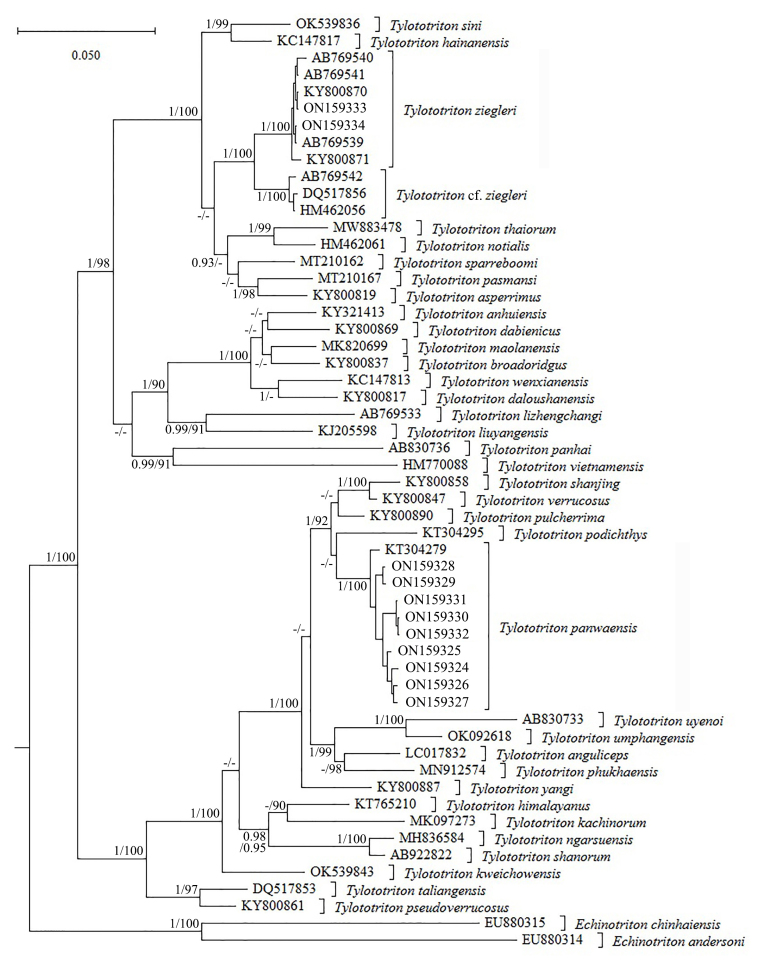
Bayesian Inference tree, based on mitochondrial ND2 sequences. Numbers before slashes indicate Bayesian posterior probabilities (values below 0.9 are not shown) and numbers after slashes indicate bootstrap support for Maximum Likelihood analyses (values below 90 are not shown).

The obtained sequence alignment is 1044 bp in length. BI and ML analyses showed basically consistent topology (Fig. 4). The two specimens collected from Malipo County, Wenshan Prefecture, Yunnan, China, were homogeneous and clustered with *Tylototritonziegleri* (including the holotype) from Ha Giang Province, Vietnam, with strong support. The genetic distance (uncorrected p-distance) between the specimens from China and *T.ziegleri* (including the holotype) from Ha Giang, Vietnam was only 0.1% (Suppl. material 1).

Combining the results of morphological and molecular analysis, we determined that the specimens from Malipo County, Wenshan Prefecture, Yunnan, China, belong to *Tylototritonziegleri*.

## Discussion

In the phylogenetic analyses in Nishikawa et al. (2013) and in this study, *Tylototritonziegleri* formed two strongly-supported lineages, one (including the holotype) from Ha Giang Province, Vietnam and the other from Cao Bang Province, Vietnam and there is a relatively large genetic divergence (2.6%) between the two lineages, more than that (2.1%) between *T.verrucosus* and *T.pulcherrima* and close to that (2.7%) between *T.taliangensis* and *T.pseudoverrucosus*. Therefore, we regard the lineage containing the holotype as *T.ziegleri* sensu stricto and the other lineage as Tylototritoncf.ziegleri. The lineage of Tylototritoncf.ziegleri may, therefore, represent a cryptic new species of *Tylototriton*. Our newly-collected specimens from China clustered with the lineage of *T.ziegleri* sensu stricto and showed negligible genetic divergence (0.1%) with this lineage, confirming *T.ziegleri* sensu stricto. Ziegler et al. (2018) reported the longevity of *T.ziegleri* and extended the diagnosis of this species, based on specimens collected from Cao Bang, Vietnam; according to their result of molecular identification, these specimens from Cao Bang should be assigned to to Tylototritoncf.ziegleri rather than *T.ziegleri* sensu stricto. As for the population in Jingxi, Guangxi, China, record by Hernandez (2016), judging from the geographical location, we consider that this population should also belong to Tylototritoncf.ziegleri.

In the original description of *Tylototritonverrucosus*, Anderson (1871) described this species as uniform blackish-brown, but not orange-patterned. Usually, the uniform blackish-brown colour of these salamanders in life remains constant in preservative and the orange colour of these salamanders in life fades to light yellow or white, but the pattern remains distinct in preservative. However, according to Nussbaum et al. (1995) and Chanda et al. (2000), all the syntypes of *T.verrucosus* are orange-patterned. This has always made people wonder why the syntypes were inconsistent with the original description by Anderson (1871). Anderson (1871) did not specify a precise type locality, but merely mentioned some valleys in western Yunnan, including Nantin, Momien, Hotha, Ponsee and Nampoung. That is to say, any of these valleys may be the type locality of *T.verrucosus*. “Momien” generally refers to Tengchong City, Yunnan, China, nowadays; “Hotha” generally refers to Husa Township, Longchuan County, Yunnan, China, nowadays; “Ponsee” generally refers to Xueli Village, Yingjiang County, Yunnan, China, nowadays; “Nampoung” generally refers to Nabang Town, Yingjiang County, Yunnan, China, nowadays; and “Nantin” is uncertain. Nussbaum et al. (1995) considered that the syntypes of *T.verrucosus* are inconsistent with the original description of *T.verrucosus* and, as the specimens from Husa agree with the original description of *T.verrucosus*, they designated a neotype (KIZ 74II0061 VI.16) of *T.verrucosus* from Gongwa Village, Longchuan County, Yunnan Province, China. However, Fei et al. (2006) questioned the validity of this neotype. Since the syntypes of *T.verrucosus* are extant (Sclater 1892, Chanda et al. 2000), one of the syntypes should be selected as the lectotype instead of designating a neotype and this designation contravenes the requirements of Art. 74.7 of the Code (ICZN, 1999). Therefore, we agree with Fei et al. (2006) that the designation of the neotype (KIZ 74II0061 VI.16) is unvalid.

**Figure 5.**
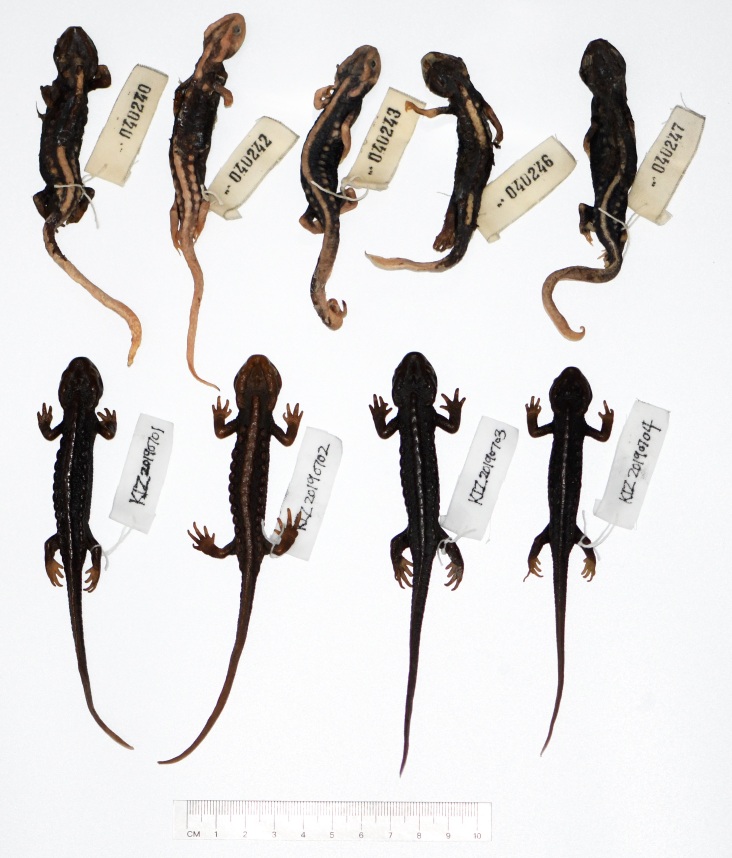
The specimens of *Tylototritonpanwaensis* from western Yunnan, China, in preservative.

We collected some specimens from western Tengchong City and neighbouring north-western Yingjiang County, Yunnan, China, in 2019; these specimens were uniform blackish-brown or uniform brown in life, that is to say, not only the specimens from Husa agree with the original description of *T.verrucosus*, but also the specimens from western Tengchong and north-western Yingjiang agree with the original description of *T.verrucosus*. Therefore, Tengchong or Yingjiang cannot be ruled out as the type locality of *T.verrucosus*. Interestingly, the black surface skins of the ones who underwent poor preservation fell off and the body colour turned to orange-patterned, while others are still uniform blackish-brown or uniform brown (Fig. 5). The colour of some of these specimens in preservative became inconsistent with that in life; this seems to be similar to the case of the syntypes of *T.verrucosus*. Nevertheless, as we do not know the previous preservation status of the syntypes of *T.verrucosus*, we cannot determine whether the colours of these syntypes have changed. More interestingly, the specimens from western Tengchong and north-western Yingjiang were assigned to *T.panwaensis* in the molecular analysis.

Although the population in Husa and the population in western Tengchong are both in agreement with the original description of *Tylototritonverrucosus*, the two populations belong to two different species. Since it is impossible to tell whether Hotha or Momien is the type locality of *T.verrucosus*, it is impossible to identify whether the population in Husa or in western Tengchong is the true *T.verrucosus*. If Hotha is the type locality of *T.verrucosus*, then the population in Husa is the true *T.verrucosus* and *T.panwaensis* is a valid species and also distributed in China; and if Momien is the type locality of *T.verrucosus*, then the population in western Tengchong may be the true *T.verrucosus*, *T.panwaensis* may be the synonym of *T.verrucosus* and the population in Husa may remain an unnamed species. We can only make this speculation at present, as more research is needed to solve this problem.

## Supplementary Material

XML Treatment for Tylototriton ziegleri

730C9316-45FF-52B4-BAEE-8AED77E510D6 10.3897/BDJ.10.e82707.suppl1 Supplementary material 1 Mean uncorrected p-distances (%), based on mitochondrial ND2 sequences. Data type Table File: oo_661732.doc https://binary.pensoft.net/file/661732 Shuo Liu, Miao Hou, Dingqi Rao

## Figures and Tables

**Table 1.** Localities, voucher information and GenBank accession numbers for all samples used in this study.

Taxa	Locality	Voucher	Accession
*Tylototritonanguliceps*	Vietnam: Dien Bien: Muong Nhe	VNMN A20143	LC017832
*Tylototritonanhuiensis*	China: Anhui: Yuexi	AHU-13-EE-006	KY321413
*Tylototritonasperrimus*	China: Guangxi: Jinxiu	CIB GX20080714	KY800819
*Tylototritonbroadoridgus*	China: Hunan: Sangzhi	CIB200084	KY800837
*Tylototritondabienicus*	China: Anhui: Shangcheng	HNNU 1004-026	KY800869
*Tylototritondaloushanensis*	China: Guizhou: Suiyang	CIBWG200600019	KY800817
*Tylototritonhainanensis*	China: Hainan: Mt Diaoluo	CIB 20081048	KC147817
*Tylototritonhimalayanus*	Nepal: Mechi: Illam	CIB 201406287	KT765210
*Tylototritonkachinorum*	Myanmar: Kachin: Indawgyi	ZMMU A5953	MK097273
*Tylototritonkweichowensis*	China: Guizhou: Shuicheng	SYS a004967	OK539843
*Tylototritonliuyangensis*	China: Hunan: Liuyang	CSUFT 20100108	KJ205598
*Tylototritonlizhengchangi*	China: Hunan: Yizhang	KUHE 42317	AB769533
*Tylototritonmaolanensis*	China: Guizhou: Libo	CIBML20180427001	MK820699
*Tylototritonngarsuensis*	Myanmar: Shan: Taunggyi	LSUHC 13763	MH836584
*Tylototritonnotialis*	Laos: Khammouan: Boualapha	FMNH HERP 271120	HM462061
*Tylototritonpanhai*	Thailand: Loei: Phu Luang WS	PL009	AB830736
*Tylototritonpanwaensis*	Myanmar: Kachin: Myitkyina	CAS 245418	KT304279
*Tylototritonpanwaensis*	China: Yunnan: Tengchong	KIZ 040240	ON159332
*Tylototritonpanwaensis*	China: Yunnan: Tengchong	KIZ 040242	ON159331
*Tylototritonpanwaensis*	China: Yunnan: Tengchong	KIZ 040243	ON159330
*Tylototritonpanwaensis*	China: Yunnan: Tengchong	KIZ 040246	ON159329
*Tylototritonpanwaensis*	China: Yunnan: Tengchong	KIZ 040247	ON159328
*Tylototritonpanwaensis*	China: Yunnan: Yingjiang	KIZ20190701	ON159327
*Tylototritonpanwaensis*	China: Yunnan: Yingjiang	KIZ20190702	ON159326
*Tylototritonpanwaensis*	China: Yunnan: Yingjiang	KIZ20190703	ON159325
*Tylototritonpanwaensis*	China: Yunnan: Yingjiang	KIZ20190704	ON159324
*Tylototritonpasmansi*	Vietnam: Phu Tho: Tan Son	IEBR 4467	MT210167
*Tylototritonphukhaensis*	Thailand: Nan: Doi Phu Kha NP	CUMZ A-7718	MN912574
*Tylototritonpodichthys*	Laos: Luang Phabang: Phoukhoun	NCSM 77725	KT304295
*Tylototritonpseudoverrucosus*	China: Sichuan: Ningnan	CIB WCG2012003	KY800861
*Tylototritonpulcherrima*	China: Yunnan: Lüchun	CIB TY040	KY800890
*Tylototritonshanjing*	China: Yunnan: Jingdong	KIZ 201306102	KY800858
*Tylototritonshanorum*	Myanmar: Shan: Taunggyi	CAS 230933	AB922822
*Tylototritonsini*	China: Guangdong: Mt Yunkai	SYS a008354	OK539836
*Tylototritonsparreboomi*	Sin Ho, Lai Chau, Vietnam	IEBR 4476	MT210162
*Tylototritontaliangensis*	China: Sichuan: Liangshan	CAS 195126	DQ517853
*Tylototritonthaiorum*	Vietnam: Nghe An: Pu Hoat NR	ZMMU A-7577	MW883478
*Tylototritonumphangensis*	Thailand: Tak: Umphang WS	CUMZ-A-8243	OK092618
*Tylototritonuyenoi*	Thailand: Chiang Mai: Doi Suthep	KUHE 19147	AB830733
*Tylototritonverrucosus*	China: Yunnan: Longchuan	CIB TSHS1	KY800847
*Tylototritonvietnamensis*	Vietnam: Bac Giang: Son Dong	IEBR 3243	HM770088
*Tylototritonwenxianensis*	China: Gansu: Wenxian	CIB 20090527	KC147813
*Tylototritonyangi*	China: Yunnan: Pingbian	KUHE 42282	KY800887
*Tylototritonziegleri*	Vietnam: Ha Giang: Quan Ba	VNMN 3390	AB769539
*Tylototritonziegleri*	Vietnam: Ha Giang: Quan Ba	KUHE 55077	AB769540
*Tylototritonziegleri*	Vietnam: Ha Giang: Quan Ba	KUHE 55078	AB769541
*Tylototritonziegleri*	Vietnam: Ha Giang: Quan Ba	VNUH HG.081	KY800871
*Tylototritonziegleri*	Vietnam: Ha Giang: Quan Ba	VNUH HG.082	KY800870
*Tylototritonziegleri*	China: Yunnan: Malipo	KIZ20210504	ON159334
*Tylototritonziegleri*	China: Yunnan: Malipo	KIZ20210505	ON159333
Tylototritoncf.ziegleri	Vietnam: Cao Bang: Bao Lac	VNMN 3389	AB769542
Tylototritoncf.ziegleri	Vietnam: Cao Bang: Quang Thanh	ROM 35330	DQ517856
Tylototritoncf.ziegleri	Vietnam: Cao Bang: Quang Thanh	ROM 35364	HM462056
*Echinotritonchinhaiensis*	China: Zhejiang: Ningbo	TP26195	EU880315
*Echinotritonandersoni*	Japan: Kagoshima: Tokunoshima	MVZ 232187	EU880314

**Table 2.** Measurements (in mm) of the specimens of *Tylototritonziegleri* from China.

	KIZ20210504	KIZ20210505
SVL	58.2	60.8
HL	15.3	15.7
HW	16.0	15.1
MXHW	16.0	15.2
SL	5.8	6.3
LJL	12.7	12.6
ENL	3.7	3.8
IND	5.4	6.1
IOD	8.6	8.6
UEW	1.6	1.6
UEL	3.3	3.2
OL	4.2	4.2
AGD	28.9	31.6
TRL	43.2	45.8
TAL	69.7	68.0
VL	7.1	5.9
BTAW	5.5	6.0
MTAW	2.7	3.0
BTAH	7.4	7.2
MXTAH	7.4	7.3
MTAH	6.9	7.0
FLL	19.5	21.8
HLL	22.5	24.0
2FL	3.7	4.2
3FL	4.0	4.5
3TL	5.5	5.3
5TL	2.3	2.4

## References

[B7700174] Anderson J (1871). Description of a new genus of newts from western Yunnan. Proceedings of the Zoological Society of London.

[B7700362] Chanda SK, Das I, Dubois A (2000). Catalogue of amphibian types in the collection of the Zoological Survey of India. Hamadryad.

[B7700104] Edgar R. C. (2004). MUSCLE: multiple sequence alignment with high accuracy and high throughput. Nucleic Acids Research.

[B7794343] Fei L, Hu SQ, Ye CY, Huang YZ (2006). Fauna Sinica. Amphibia. Volume 1. General Accounts of *Gymnophiona* and *Urodela*.

[B7794335] Hernandez Axel (2016). Crocodile Newts: The Primitive Salamandridae of Asia: Genera *Echinotriton* and *Tylototriton*.

[B7700113] Kalyaanamoorthy Subha, Minh Bui Quang, Wong Thomas K F, von Haeseler Arndt, Jermiin Lars S (2017). ModelFinder: fast model selection for accurate phylogenetic estimates. Nature Methods.

[B7700040] Li H, Yang B, Chi H, Lai R, Liu C, Xu Z, Zhu X, Gong S, Chen J (2020). Distribution and supplementary description of *Tylototritonziegleri* in China. Chinese Journal of Wildlife.

[B7700054] Lyu Zhi-Tong, Wang Jian, Zeng Zhao-Chi, Zhou Jia-Jun, Qi Shuo, Wan Han, Li You-Yu, Wang Ying-Yong (2021). A new species of the genus *Tylototriton* (Caudata, Salamandridae) from Guangdong, southern China, with discussion on the subgenera and species groups within the genus. Vertebrate Zoology.

[B7700138] Nguyen Lam-Tung, Schmidt Heiko A., von Haeseler Arndt, Minh Bui Quang (2015). IQ-TREE: A fast and effective stochastic algorithm for estimating maximum-likelihood phylogenies. Molecular Biology and Evolution.

[B7700008] Nishikawa Kanto, Matsui Masafumi, Nguyen Tao Thien (2013). A new species of *Tylototriton* from Northern Vietnam (Amphibia: Urodela: Salamandridae). Current Herpetology.

[B7700165] Nussbaum RA, Brodie Jr, ED, Yang DT (1995). A taxonomic review of *Tylototritonverrucosus* Anderson (Amphibia: Caudata: Salamandridae). Herpetologica.

[B7700123] Ronquist Fredrik, Teslenko Maxim, van der Mark Paul, Ayres Daniel L., Darling Aaron, Höhna Sebastian, Larget Bret, Liu Liang, Suchard Marc A., Huelsenbeck John P. (2012). MrBayes 3.2: Efficient Bayesian phylogenetic inference and model choice across a large model space. Systematic Biology.

[B7700067] Sambrook J, Fritsch E, Maniatis T (1989). Molecular cloning: a laboratory manual. 2nd Edition. Vol. 1–3.

[B7700339] Sclater WL (1892). A list of the Batrachia of the Indian Museum.

[B7700147] Tamura Koichiro, Stecher Glen, Kumar Sudhir (2021). MEGA11: Molecular Evolutionary Genetics Analysis version 11. Molecular Biology and Evolution.

[B7700026] Ye Jiang, Wei Zhimin, Han Fuyao, Ni Qingyong, Yao Yongfang, Xu Huailiang, Li Ying, Rao Dingqi, Zhang Mingwang (2017). The complete mitogenome sequence of *Tylototritonziegleri* (Amphibia: Caudata). Conservation Genetics Resources.

[B7794351] Ziegler Thomas, Marcec Ruth, Vardukyan David, Nguyen Truong Quang, Le Minh Duc, Bernardes Marta (2018). First record of longevity in Ziegler’s crocodile newt, *Tylototritonziegleri* Nishikawa, Matsui & Nguyen, 2013 (Caudata, Salamandridae). Alytes.

