# Supplementary material for: Confirmation of Tylototritonziegleri Nishikawa, Matsui & Nguyen, 2013 in China, with discussion on the relationship between T.verrucosus Anderson, 1871 and T.panwaensis Grismer, Wood, Quah, Thura, Espinoza & Murdoch, 2019 (Caudata, Salamandridae)
Source: Biodivers Data J. 2022 May 19;10:e82707. doi: 10.3897/BDJ.10.e82707 (PMC9848457; doi:10.3897/BDJ.10.e82707)
Supplement: Supplementary material 1 — Mean uncorrected p-distances (%), based on mitochondrial ND2 sequences. [file bdj-10-e82707-s001.doc]

**Table S1.** Mean uncorrected p-distances (%), based on mitochondrial ND2 sequences.

|  |  | 1 | 2 | 3 | 4 | 5 | 6 | 7 | 8 | 9 | 10 | 11 | 12 | 13 | 14 | 15 | 16 | 17 | 18 | 19 | 20 | 21 | 22 | 23 | 24 | 25 | 26 | 27 | 28 | 29 | 30 | 31 | 32 | 33 | 34 | 35 | 36 | 37 | 38 | 39 |
| --- | --- | --- | --- | --- | --- | --- | --- | --- | --- | --- | --- | --- | --- | --- | --- | --- | --- | --- | --- | --- | --- | --- | --- | --- | --- | --- | --- | --- | --- | --- | --- | --- | --- | --- | --- | --- | --- | --- | --- | --- |
| 1 | *Tylototriton anguliceps* |  |  |  |  |  |  |  |  |  |  |  |  |  |  |  |  |  |  |  |  |  |  |  |  |  |  |  |  |  |  |  |  |  |  |  |  |  |  |  |
| 2 | *Tylototriton anhuiensis* | 10.7 |  |  |  |  |  |  |  |  |  |  |  |  |  |  |  |  |  |  |  |  |  |  |  |  |  |  |  |  |  |  |  |  |  |  |  |  |  |  |
| 3 | *Tylototriton asperrimus* | 11.4 | 8.1 |  |  |  |  |  |  |  |  |  |  |  |  |  |  |  |  |  |  |  |  |  |  |  |  |  |  |  |  |  |  |  |  |  |  |  |  |  |
| 4 | *Tylototriton broadoridgus* | 11.0 | 3.5 | 8.5 |  |  |  |  |  |  |  |  |  |  |  |  |  |  |  |  |  |  |  |  |  |  |  |  |  |  |  |  |  |  |  |  |  |  |  |  |
| 5 | *Tylototriton dabienicus* | 12.1 | 3.7 | 9.0 | 3.4 |  |  |  |  |  |  |  |  |  |  |  |  |  |  |  |  |  |  |  |  |  |  |  |  |  |  |  |  |  |  |  |  |  |  |  |
| 6 | *Tylototriton daloushanensis* | 11.8 | 4.4 | 9.3 | 4.3 | 4.4 |  |  |  |  |  |  |  |  |  |  |  |  |  |  |  |  |  |  |  |  |  |  |  |  |  |  |  |  |  |  |  |  |  |  |
| 7 | *Tylototriton hainanensis* | 10.0 | 7.8 | 5.0 | 7.6 | 8.8 | 9.2 |  |  |  |  |  |  |  |  |  |  |  |  |  |  |  |  |  |  |  |  |  |  |  |  |  |  |  |  |  |  |  |  |  |
| 8 | *Tylototriton himalayanus* | 6.8 | 10.7 | 11.6 | 10.5 | 11.3 | 11.1 | 9.9 |  |  |  |  |  |  |  |  |  |  |  |  |  |  |  |  |  |  |  |  |  |  |  |  |  |  |  |  |  |  |  |  |
| 9 | *Tylototriton kachinorum* | 7.7 | 10.6 | 13.3 | 11.3 | 11.7 | 11.7 | 11.9 | 5.3 |  |  |  |  |  |  |  |  |  |  |  |  |  |  |  |  |  |  |  |  |  |  |  |  |  |  |  |  |  |  |  |
| 10 | *Tylototriton kweichowensis* | 6.4 | 9.7 | 10.3 | 9.0 | 10.4 | 10.6 | 8.6 | 5.4 | 6.4 |  |  |  |  |  |  |  |  |  |  |  |  |  |  |  |  |  |  |  |  |  |  |  |  |  |  |  |  |  |  |
| 11 | *Tylototriton liuyangensis* | 10.3 | 7.1 | 8.8 | 7.1 | 7.3 | 7.5 | 7.8 | 10.0 | 12.4 | 9.6 |  |  |  |  |  |  |  |  |  |  |  |  |  |  |  |  |  |  |  |  |  |  |  |  |  |  |  |  |  |
| 12 | *Tylototriton lizhengchangi* | 12.3 | 8.1 | 10.4 | 8.4 | 8.7 | 8.6 | 8.7 | 11.5 | 14.6 | 10.8 | 7.1 |  |  |  |  |  |  |  |  |  |  |  |  |  |  |  |  |  |  |  |  |  |  |  |  |  |  |  |  |
| 13 | *Tylototriton maolanensis* | 11.0 | 3.3 | 8.6 | 3.0 | 3.7 | 4.3 | 8.3 | 10.7 | 11.7 | 9.7 | 7.3 | 8.1 |  |  |  |  |  |  |  |  |  |  |  |  |  |  |  |  |  |  |  |  |  |  |  |  |  |  |  |
| 14 | *Tylototriton ngarsuensis* | 6.9 | 11.3 | 11.3 | 10.9 | 12.6 | 12.0 | 10.1 | 5.9 | 7.7 | 6.7 | 11.1 | 12.1 | 11.5 |  |  |  |  |  |  |  |  |  |  |  |  |  |  |  |  |  |  |  |  |  |  |  |  |  |  |
| 15 | *Tylototriton notialis* | 10.5 | 8.3 | 4.9 | 8.4 | 9.2 | 9.5 | 4.8 | 10.7 | 11.9 | 9.8 | 8.8 | 10.0 | 8.6 | 11.2 |  |  |  |  |  |  |  |  |  |  |  |  |  |  |  |  |  |  |  |  |  |  |  |  |  |
| 16 | *Tylototriton panhai* | 13.0 | 9.5 | 11.4 | 9.5 | 9.6 | 10.2 | 9.8 | 12.3 | 13.3 | 10.7 | 9.6 | 9.9 | 10.2 | 12.4 | 10.4 |  |  |  |  |  |  |  |  |  |  |  |  |  |  |  |  |  |  |  |  |  |  |  |  |
| 17 | *Tylototriton panwaensis* (Myanmar) | 4.4 | 10.0 | 10.9 | 10.0 | 10.4 | 10.5 | 10.0 | 6.6 | 6.4 | 6.0 | 10.0 | 11.4 | 10.0 | 6.3 | 10.9 | 12.6 |  |  |  |  |  |  |  |  |  |  |  |  |  |  |  |  |  |  |  |  |  |  |  |
| 18 | *Tylototriton panwaensis* (China) | 4.4 | 9.9 | 10.9 | 10.0 | 10.5 | 10.4 | 10.0 | 6.8 | 6.8 | 6.1 | 10.2 | 11.3 | 10.0 | 6.4 | 10.7 | 12.5 | 0.8 |  |  |  |  |  |  |  |  |  |  |  |  |  |  |  |  |  |  |  |  |  |  |
| 19 | *Tylototriton pasmansi* | 11.5 | 8.2 | 3.4 | 8.3 | 9.3 | 9.6 | 4.8 | 11.3 | 13.3 | 10.2 | 8.4 | 10.0 | 9.0 | 12.0 | 4.6 | 10.7 | 11.3 | 11.0 |  |  |  |  |  |  |  |  |  |  |  |  |  |  |  |  |  |  |  |  |  |
| 20 | *Tylototriton phukhaensis* | 4.0 | 11.2 | 11.8 | 11.3 | 12.3 | 12.3 | 10.6 | 7.2 | 8.6 | 6.5 | 10.3 | 12.5 | 11.1 | 8.0 | 11.0 | 13.0 | 5.4 | 5.5 | 12.1 |  |  |  |  |  |  |  |  |  |  |  |  |  |  |  |  |  |  |  |  |
| 21 | *Tylototriton podichthys* | 5.3 | 9.7 | 10.8 | 10.0 | 10.8 | 10.1 | 10.1 | 6.9 | 8.0 | 6.2 | 10.0 | 11.2 | 10.0 | 8.1 | 10.6 | 12.2 | 3.7 | 3.8 | 11.2 | 6.0 |  |  |  |  |  |  |  |  |  |  |  |  |  |  |  |  |  |  |  |
| 22 | *Tylototriton pseudoverrucosus* | 8.6 | 9.3 | 9.7 | 9.3 | 9.7 | 9.7 | 8.5 | 7.1 | 7.3 | 6.0 | 9.2 | 10.0 | 9.3 | 8.7 | 9.3 | 10.5 | 7.5 | 7.5 | 9.4 | 8.2 | 7.2 |  |  |  |  |  |  |  |  |  |  |  |  |  |  |  |  |  |  |
| 23 | *Tylototriton pulcherrima* | 4.0 | 9.8 | 10.9 | 9.9 | 10.5 | 10.2 | 9.5 | 6.3 | 7.7 | 5.4 | 9.8 | 10.8 | 9.8 | 6.5 | 10.2 | 12.0 | 2.6 | 2.8 | 11.2 | 4.2 | 3.7 | 6.7 |  |  |  |  |  |  |  |  |  |  |  |  |  |  |  |  |  |
| 24 | *Tylototriton shanjing* | 4.5 | 10.3 | 11.6 | 10.1 | 11.1 | 10.7 | 10.3 | 6.3 | 8.0 | 6.2 | 10.7 | 11.8 | 10.2 | 6.7 | 11.1 | 12.9 | 3.3 | 3.4 | 11.9 | 5.2 | 4.5 | 7.8 | 2.6 |  |  |  |  |  |  |  |  |  |  |  |  |  |  |  |  |
| 25 | *Tylototriton shanorum* | 6.9 | 10.7 | 11.6 | 10.3 | 11.2 | 11.4 | 10.2 | 5.3 | 8.0 | 6.3 | 10.5 | 12.0 | 10.4 | 1.2 | 10.7 | 12.5 | 6.7 | 6.8 | 11.4 | 8.1 | 8.0 | 8.3 | 6.7 | 6.8 |  |  |  |  |  |  |  |  |  |  |  |  |  |  |  |
| 26 | *Tylototriton sini* | 9.5 | 8.0 | 5.2 | 8.6 | 9.0 | 8.8 | 3.2 | 9.6 | 12.2 | 9.0 | 7.9 | 9.0 | 8.5 | 9.6 | 5.5 | 10.4 | 9.6 | 9.4 | 5.1 | 10.4 | 9.6 | 8.5 | 9.3 | 10.1 | 10.0 |  |  |  |  |  |  |  |  |  |  |  |  |  |  |
| 27 | *Tylototriton sparreboomi* | 10.8 | 7.7 | 4.2 | 8.0 | 8.5 | 9.3 | 4.4 | 10.6 | 11.7 | 9.8 | 8.7 | 9.8 | 8.6 | 11.5 | 4.7 | 10.2 | 11.0 | 10.9 | 4.0 | 11.3 | 10.9 | 9.1 | 10.3 | 11.5 | 10.8 | 4.8 |  |  |  |  |  |  |  |  |  |  |  |  |  |
| 28 | *Tylototriton taliangensis* | 8.4 | 8.8 | 9.3 | 9.3 | 9.3 | 9.5 | 8.6 | 7.6 | 7.3 | 6.4 | 9.1 | 9.5 | 9.1 | 8.9 | 9.4 | 10.6 | 7.7 | 7.4 | 9.8 | 8.3 | 7.7 | 2.7 | 7.2 | 7.5 | 8.4 | 8.7 | 9.3 |  |  |  |  |  |  |  |  |  |  |  |  |
| 29 | *Tylototriton thaiorum* | 10.6 | 8.0 | 5.3 | 7.9 | 8.7 | 9.0 | 4.6 | 10.6 | 12.8 | 9.9 | 8.6 | 9.6 | 8.1 | 12.0 | 2.8 | 10.5 | 10.7 | 10.6 | 4.6 | 10.8 | 10.6 | 8.9 | 10.2 | 11.1 | 11.0 | 5.8 | 4.6 | 9.0 |  |  |  |  |  |  |  |  |  |  |  |
| 30 | *Tylototriton umphangensis* | 5.0 | 12.2 | 12.2 | 12.6 | 13.2 | 13.1 | 11.6 | 7.9 | 8.8 | 8.1 | 12.0 | 13.2 | 12.5 | 8.4 | 11.7 | 13.9 | 5.4 | 5.6 | 12.2 | 5.8 | 7.1 | 9.5 | 5.5 | 5.2 | 8.3 | 11.5 | 12.1 | 9.2 | 11.8 |  |  |  |  |  |  |  |  |  |  |
| 31 | *Tylototriton uyenoi* | 7.3 | 12.6 | 12.9 | 12.8 | 13.2 | 13.2 | 12.5 | 8.6 | 11.7 | 8.1 | 12.7 | 12.9 | 12.6 | 8.8 | 13.0 | 14.2 | 7.6 | 7.6 | 13.0 | 7.3 | 8.2 | 10.0 | 6.8 | 7.5 | 9.4 | 12.3 | 13.6 | 10.0 | 12.7 | 4.9 |  |  |  |  |  |  |  |  |  |
| 32 | *Tylototriton verrucosus* | 4.4 | 10.4 | 11.5 | 10.2 | 11.0 | 10.8 | 10.2 | 6.4 | 7.3 | 5.9 | 10.2 | 11.5 | 10.1 | 6.5 | 11.0 | 12.7 | 2.4 | 2.7 | 11.8 | 4.7 | 4.1 | 7.5 | 2.1 | 1.3 | 6.5 | 10.0 | 11.4 | 7.4 | 11.0 | 4.9 | 7.2 |  |  |  |  |  |  |  |  |
| 33 | *Tylototriton vietnamensis* | 13.1 | 10.4 | 10.8 | 10.9 | 10.6 | 11.1 | 10.5 | 12.5 | 12.8 | 11.7 | 10.8 | 11.6 | 11.0 | 13.0 | 11.3 | 11.1 | 11.8 | 12.1 | 11.4 | 13.6 | 12.9 | 11.5 | 12.0 | 12.5 | 12.6 | 10.8 | 10.4 | 11.0 | 11.6 | 13.4 | 14.8 | 12.0 |  |  |  |  |  |  |  |
| 34 | *Tylototriton wenxianensis* | 10.9 | 4.1 | 8.8 | 4.3 | 4.8 | 3.9 | 8.8 | 10.7 | 11.5 | 9.9 | 7.1 | 8.0 | 4.3 | 11.9 | 9.0 | 10.3 | 9.7 | 9.6 | 9.6 | 11.2 | 9.7 | 9.4 | 9.2 | 10.0 | 11.1 | 9.2 | 9.2 | 8.7 | 8.7 | 12.3 | 12.6 | 9.8 | 10.4 |  |  |  |  |  |  |
| 35 | *Tylototriton yangi* | 4.2 | 10.0 | 10.1 | 10.0 | 11.0 | 10.4 | 9.6 | 6.5 | 8.2 | 6.4 | 9.4 | 11.0 | 9.8 | 6.4 | 10.2 | 12.6 | 4.1 | 4.1 | 10.5 | 5.4 | 5.1 | 7.4 | 3.8 | 4.6 | 6.9 | 8.8 | 10.0 | 7.8 | 10.3 | 5.6 | 7.5 | 4.0 | 12.1 | 9.8 |  |  |  |  |  |
| 36 | *Tylototriton ziegleri* (Vietnam) | 11.0 | 8.0 | 4.6 | 8.0 | 8.9 | 9.4 | 4.4 | 11.1 | 11.8 | 9.6 | 8.6 | 10.1 | 8.6 | 11.7 | 5.0 | 10.0 | 10.6 | 10.6 | 4.9 | 11.3 | 10.8 | 9.3 | 10.5 | 11.3 | 11.2 | 5.3 | 4.5 | 9.5 | 4.9 | 12.2 | 13.4 | 11.2 | 11.1 | 9.0 | 10.1 |  |  |  |  |
| 37 | *Tylototriton ziegleri* (China) | 10.9 | 7.9 | 4.4 | 7.9 | 8.8 | 9.3 | 4.3 | 11.0 | 11.7 | 9.6 | 8.5 | 10.0 | 8.5 | 11.6 | 5.0 | 10.0 | 10.5 | 10.5 | 4.8 | 11.2 | 10.7 | 9.3 | 10.4 | 11.3 | 11.2 | 5.3 | 4.3 | 9.5 | 4.9 | 12.1 | 13.3 | 11.2 | 11.1 | 8.9 | 10.0 | 0.1 |  |  |  |
| 38 | *Tylototriton* cf. *ziegleri* | 11.0 | 8.6 | 4.6 | 8.4 | 9.4 | 9.9 | 4.3 | 11.5 | 11.7 | 9.9 | 8.3 | 9.9 | 9.0 | 12.0 | 5.0 | 10.1 | 10.8 | 10.5 | 4.7 | 11.5 | 10.9 | 9.4 | 10.6 | 11.6 | 11.5 | 4.8 | 4.3 | 10.0 | 5.2 | 12.2 | 13.6 | 11.5 | 11.3 | 9.5 | 10.2 | 2.6 | 2.5 |  |  |
| 39 | *Echinotriton andersoni* | 16.1 | 14.2 | 14.5 | 13.8 | 14.6 | 14.8 | 13.4 | 15.4 | 19.7 | 15.3 | 14.2 | 14.5 | 15.2 | 13.1 | 14.8 | 15.2 | 14.5 | 14.6 | 14.1 | 17.0 | 15.4 | 14.6 | 15.1 | 15.3 | 15.0 | 13.5 | 14.1 | 14.5 | 14.3 | 16.7 | 17.2 | 15.3 | 15.8 | 14.8 | 15.4 | 14.4 | 14.2 | 14.1 |  |
| 40 | *Echinotriton chinhaiensis* | 14.7 | 13.2 | 14.2 | 13.3 | 13.4 | 13.8 | 13.7 | 14.4 | 16.6 | 13.9 | 12.9 | 13.6 | 14.4 | 13.2 | 13.6 | 14.6 | 14.5 | 14.3 | 14.4 | 15.8 | 14.7 | 13.2 | 14.5 | 15.0 | 14.4 | 13.7 | 13.8 | 13.2 | 13.5 | 16.5 | 17.2 | 15.0 | 14.5 | 13.9 | 14.2 | 13.9 | 13.8 | 14.0 | 12.2 |
